# Bulk and surface topological indices for a skyrmion string: current-driven dynamics of skyrmion string in stepped samples

**DOI:** 10.1038/s41598-020-76469-5

**Published:** 2020-11-20

**Authors:** Wataru Koshibae, Naoto Nagaosa

**Affiliations:** 1 RIKEN Center for Emergent Matter Science (CEMS), Wako, Saitama 351-0198 Japan; 2 Department of Applied Physics, The University of Tokyo, 7-3-1, Hongo, Bunkyo-ku, Tokyo, 113-8656 Japan

**Keywords:** Spintronics, Topological matter

## Abstract

The magnetic skyrmion is a topological magnetic vortex, and its topological nature is characterized by an index called skyrmion number which is a mapping of the magnetic moments defined on a two-dimensional space to a unit sphere. In three-dimensions, a skyrmion, i.e., a vortex penetrating though the magnet naturally forms a string, which terminates at the surfaces of the magnet or in the bulk. For such a string, the topological indices, which control its topological stability are less trivial. Here, we study theoretically, in terms of numerical simulation, the dynamics of current-driven motion of a skyrmion string in a film sample with the step edges on the surface. In particular, skyrmion–antiskyrmion pair is generated by driving a skyrmion string through the side step with an enough height. We find that the topological indices relevant to the stability are the followings; (1) skyrmion number along the developed surface, and (2) the monopole charge in the bulk defined as the integral over the surface enclosing a singular magnetic configuration. As long as the magnetic configuration is slowly varying, the former is conserved while its changes is associated with nonzero monopole charge. The skyrmion number and the monoplole charge offer a coherent understanding of the stability of the topological magnetic texture and the nontrivial dynamics of skyrmion strings.

## Introduction

Magnetic skyrmion, a swirling magnetic vortex has attracted much attention in recent years^1–10^. The main focus is on its topological nature: the skyrmion is topologically distinguished from ferromagnetic state for instance, i.e., these magnetic textures cannot be related to each other within continuous deformation. This topological difference is characterized by the skyrmion number $N_{\text{ sk }}$. To make the definition of the index $N_{\text{ sk }}$ clear, for given normalized magnetic moments $\{\varvec{n}_{\varvec{r}}\}_{\varvec{r}\in \Lambda }$ on the set of lattice sites $\Lambda$, we define

1 $$\begin{aligned} N_{\text{ topol }}(\Omega )=&\frac{1}{2\pi }\int _{\Omega } b_{\text {normal}} d\omega , \end{aligned}$$

where $b_{\text {normal}}=\varvec{b}\cdot \varvec{e}$ with the emergent *b*-field $b_i=(1/4)\varepsilon _{ijk}\varvec{n}\cdot (\partial _j\varvec{n}\times \partial _k\varvec{n})$^11–13^ and $\varvec{e}$ is the normal unit vector to the two-dimensional domain of integral $\Omega \subset \Lambda$. (This $N_{\text{ topol }}$ is a functional of $\{\varvec{n}_{\varvec{r}}\}_{\varvec{r}\in \Omega }$ and depends on time for the dynamics, but we will not explicitly write those degrees of freedom in the expression Eq. (1).) Usually, the skyrmion number is defined as $N_{{\text{ sk}},\Omega }=N_{\text{ topol }}(\Omega )$ where $\Omega$ is a plane perpendicular to the external magnetic field and the direction ${\varvec{e}}$ is taken to be parallel to the magnetic field. Under the condition where $\varvec{n}_{\varvec{r}}\rightarrow {\varvec{e}}$ for $|\varvec{r}|\rightarrow \infty$, $N_{{\text{ sk }},\Omega }=-1$ for a skyrmion on $\Omega$.

**Figure 1:**
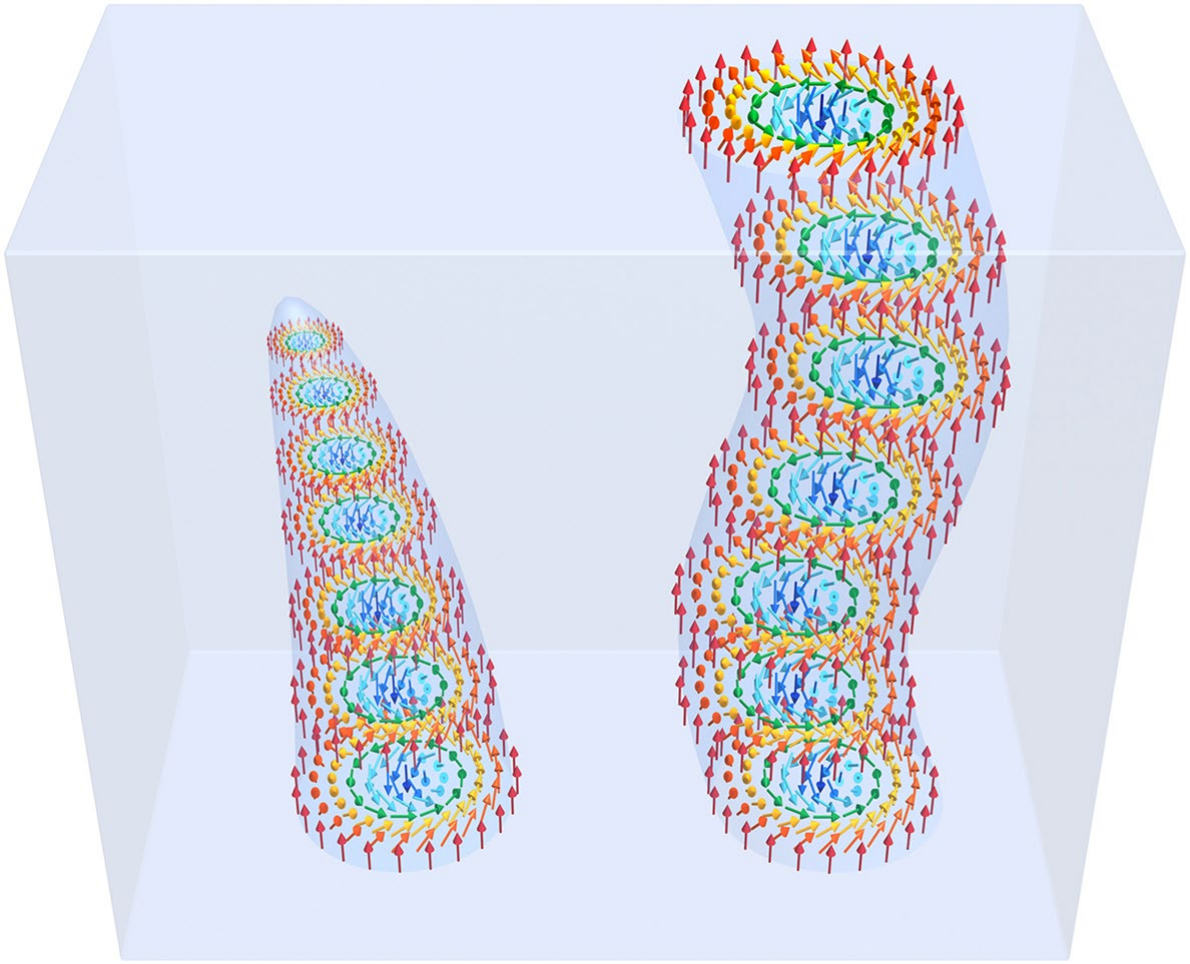
Skyrmion string ending with the skyrmion on the surface (right) and that terminates at the monopole (left). This figure is made by Mari Ishida. (See also “Supplementary Information” and Supplementary Movie S8.avi for the magnetic texture of a magnetic monopole.).

In the three dimensional magnets, the skyrmion usually forms rod-like object along the external magnetic field^14–25^. When the meandering degree of freedom is introduced, it is better to consider it as skyrmion string. When the skyrmion string terminates or branches into two skyrmion strings in the bulk, the singular points appear. (Figure 1 is a schematic for the skyrmion string (right) and that with a singular point (left).) The study of such singular points goes back over more than a half-century^26–30^. In those earlier studies^26–29^, the Bloch point, namely, the topological defect on the Bloch line was extensively studied. The topologically the same defects are sometimes called (anti)hedgehog or (anti)monopole^14,16,20–22,30^. In the present paper, we use the word, (anti)monopole, to express the topological defect on the skyrmion string.

Kotiuga^31,32^ described the topological nature of (anti)monopole by the Hopf extension theorem of algebraic topology. (The references^33,34^ give a more extensive discussion on the group theoretical description of topological matters.) It is nothing but the Gauss’ low for the topological charge and flux: the (anti)monopole is characterized by the topological index $N_{\text{ mp }}$ called monopole charge. This $N_{\text{ mp }}$ is defined by the integral of the solid angle formed by the magnetic moments over the surface enclosing the (anti)monopole: Using Eq. (1), the monopole charge is defined as $N_{\text{ mp }}=N_{\text{ topol }}(\Omega _{\varvec{r}_{\text {mp}}})=+1$ ($N_{\text{ mp }}=N_{\text{ topol }}(\Omega _{\varvec{r}_{\text {amp}}})=-1$) for $\Omega _{\varvec{r}_{\text {mp}}}$ ($\Omega _{\varvec{r}_{\text {amp}}}$) enclosing a monopole at $\varvec{r}_{\text {mp}}$ (an antimonopole at $\varvec{r}_{\text {amp}}$) with ${\varvec{e}}$ pointing outward the domain of integral. For a closed surface $\Omega$ which does not enclose the spatial defects such as void(s), Eq. (1) gives

2 $$\begin{aligned} N_{{\text{ sk}},\Omega }&=\sum _{\varvec{r}_{\text{ mp }}}N_{\text{ mp }}(\varvec{r}_{\text{ mp }}) +\sum _{\varvec{r}_{\text{ amp }}}N_{\text{ mp }}(\varvec{r}_{\text{ amp }})\nonumber \\&=\sum _{\varvec{r}_{\text{ mp }}}+1\ \ \ +\ \ \ \sum _{\varvec{r}_{\text{ amp }}}-1, \end{aligned}$$

for (anti)monopoles enclosed in $\Omega$ and (anti)skyrmions on $\Omega$. For the flux density ${\varvec{b}}/(2\pi)$, this Gauss’ law relates the skyrmion string and the (anti)monopole, i.e., those are corresponding to the flux line and its source (sink) point. The total monopole charge for the (anti)monopoles enclosed by $\Omega$ is always the same as the total skyrmion number on the surface $\Omega$, $N_{{\text {sk}},\Omega }$.

In some cases, the endpoints of a skyrmion string on the surface of magnet might be regarded as the monopole and antimonopole. However, $N_{\text{ mp }}$ cannot be defined for the surface magnetic texture since half of the space is “vacuum” where magnetic moment is absent. In particular, the (anti)monopole point $\varvec{r}_{\text {mp}}$ ($\varvec{r}_{\text {amp}}$) defined above cannot be on the surface of magnet. On the other hand, one can define $N_{{\text{ sk}},\Omega }=N_{\text{ topol }}(\Omega )$ for the magnetic texture on the surface $\Omega$, i.e., in this case, the surface of a magnet gives a well-defined orientable two-dimensional manifold $\Omega$.

The topological nature discussed above is essential to discuss the stability of the magnetic texture. For the magnetic moments on a two-dimensional lattice, the topological stability is based on the energy scales of the excitation. For example, in the chiral magnets with the ferromagnetic interaction *J* and Dzyaloshinskii–Moriya (DM) interaction *D*^35–37^, the length scale of the skyrmion size is characterized by $\sim (J/D)a$ with the lattice constant *a*, which is much larger than *a* when $D\ll J$. This fact validates the continuum approximation, and the energy density is $\sim (D^2)/(Ja^2)$. This energy density and the skyrmion size result in the order of *J* for the energy scale of the stability for a skyrmion. Therefore, a change in $N_{\text{ sk }}$, i.e., the topological transition of magnetic texture requires an overcome of the energy barrier of the order of *J*. When a skyrmion string is broken at a point $\varvec{r}_b$ in bulk, a monopole-antimonopole pair appears at the point. In other words, at the two-dimensional cross section $\Omega$ including the broken point $\varvec{r}_b$, the skyrmion number $N_{\text{ sk},{\Omega }}$ changes. Therefore, this change also requires the overcome of the energy barrier of the order of *J*.

In the present paper, we show that the surface $N_{\text{ sk }}$ plays a crucial role together with $N_{\text{ mp }}$ for the skyrmion string stability and dynamics. To this end, we numerically investigate the current driven dynamics of the skyrmion string in the magnet with step edges on the surface. The step edges act as the pinning center of the motion of a skyrmion string, which sometimes leads to the detachment of the skyrmion from the surface or the splitting of the string into pieces. By the numerical simulation, we examine the stability of the surface (anti)skyrmion and the dynamics including (anti)skyrmion–(anti)monopole collision leading to skyrmion string annihilation. These stability and dynamical processes are well understood as two kinds of topological indices; skyrmion number $N_{\text{ sk }}$ for the surface and the monopole charge $N_{\text{ mp }}$ for the bulk.

## Results

**Figure 2:**
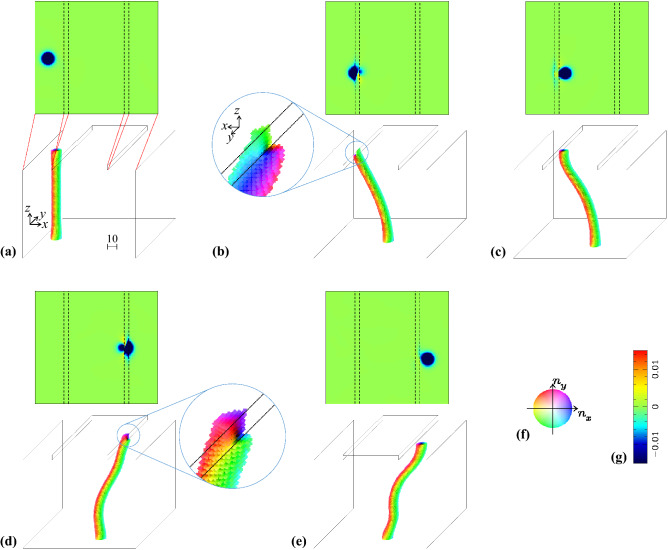
Skyrmion string in chiral magnets with step edges under an external magnetic field along *z*-direction. The case with step height 5 is shown. (**a**) The upper (lower) panel represents the spatial distribution of emergent *b*-field $b_{\text {normal}}$ normal to the surface using color code (g) [magnetic texture using color code (f)] at $t=0$. The color code (f) indicates $n_x$–$n_y$ component of the magnetic moments, e.g., blue is corresponding to the in-plane magnetic moment along *x* axis. The darkness of the color represents the $n_z$ component, i.e., black is corresponding to $\varvec{n}=(0,0,-1)$. The broken lines in the upper panel are corresponding to the edges of the upper and lower terraces as indicated by the red dotted lines. In the same way, the snapshots at (**b**) $t=6000$, (**c**) $t=8040$, (**d**) $t=11060$ and (**e**) $t=11500$ are shown. In (**b**) and (**d**), the enlarged magnetic textures at the step edges are shown. (For (**b**), the arrangement of the magnetic texture is seen from the left side.) To make the visualization of string clear, the magnetic moments with $n_z>0.5$ are not shown for the panels of magnetic texture.

To study the topological stability of (anti)skyrmion, (anti)monopole and skyrmion string, we start with a metastable skyrmion string in a three-dimensional chiral magnet with step edges (see Fig. 2). The Hamiltonian is given by

3 $$\begin{aligned} \mathcal {H}=\sum _{\varvec{r}\in \Lambda }E(\varvec{r}), \end{aligned}$$

with

4 $$\begin{aligned} E(\varvec{r})=&\sum _{\varvec{r}+{\varvec{\rho }} \in \Lambda } \frac{1}{2}\left[ -J{\varvec{n}}_{\varvec{r}} \cdot {\varvec{n}}_{{\varvec{r}}+{\varvec{\rho }}} +D\left( {\varvec{n}}_{\varvec{r}} \times {\varvec{n}}_{{\varvec{r}}+{\varvec{\rho }}} \right) \cdot {{\varvec{\rho }}} \right] \nonumber \\&-h n_{z,\varvec{r}}, \end{aligned}$$

where ${\varvec{\rho }}=\pm \hat{\varvec{x}}, \pm \hat{\varvec{y}}, \pm \hat{\varvec{z}}$ with the unit vectors $\hat{\varvec{x}}$, $\hat{\varvec{y}}$ and $\hat{\varvec{z}}$ in *x*-, *y*- and *z*-axes, and $\Lambda$ is the set for the cubic lattice sites of the system. In the lattice model, the topological stability is always related to the energy barrier and is a matter of quantitative problem. However, when the magnetic textures is spatially slowly varying, the distinction between the continuous magnetic configurations and singular ones is rather clear. The normalized magnetic moments at $\varvec{r}\in \Lambda$ is denoted by $\varvec{n}_{\varvec{r}}=(n_{x,\varvec{r}},n_{y,\varvec{r}},n_{z,\varvec{r}})$. The lattice constant is taken as the unit of length. As shown in Fig. 2, the step edges are introduced on the top surface of the magnet while the bottom surface is flat. The step edge is perpendicular to *x*-direction. In *x*- and *y*-directions, the periodic boundary condition is imposed. But, where the bottom surface and the top surface with step edges face to “vacuum”, the open boundary condition is employed. For simulations, we use the system size with $120\times 120$ for the bottom surface area and $z=1\sim 100$ at the higher terrace area. The higher terrace has a width 60. Figure 2 shows a case with a step height 5, i.e., the lower terrace is on the layer with $z=95$.

Here, we use a parameter set $\{J=1, D=0.2, h=0.06\}$ (i.e., *J* is the unit of *D* and *h*) where the ferromagnetic state polarized in *z* direction is the ground state^38,39^. Figure 2a is the relaxed metastable state with a skyrmion string which is in the lower terrace area. The skyrmion string has a tensile strain due to the metastablity, i.e., the longer string costs more energy. Therefore, the relaxed string is straight along *z* direction. Consequently, the string in the higher terrace area has an energy cost compared to the string in the lower area. In other words, the height profile of this system roughly indicates the potential profile for the skyrmion string. (See also “Supplementary Information”.)

We drive the skyrmion string by the spin–transfer–torque (STT) effect^1^:

The Landau–Lifshitz–Gilbert (LLG) equation is given by

5 $$\frac{{\text{ d }} {\varvec{n}}_{\varvec{r}}}{{\text{ d }} t}=- \frac{\partial \mathcal {H}}{\partial {\varvec{n}}_{\varvec{r}}} \times {\varvec{n}}_{\varvec{r}} +\alpha {\varvec{n}}_{\varvec{r}} \times \frac{{\text{ d }}{\varvec{n}}_{\varvec{r}}}{{\text{ d }} t} -\left( \varvec{j}\cdot \nabla \right) \varvec{n}_{\varvec{r}} +\beta \left[ \varvec{n}_{\varvec{r}}\times \left( \varvec{j}\cdot \nabla \right) \varvec{n}_{\varvec{r}}\right] ,$$

where $\alpha$ is the Gilbert damping constant. The last two terms in Eq. (5) represent the STT effect due to the spin polarlized electric current density $\varvec{j}$ with the coefficient of the non-adiabatic effect $\beta$. In the following, we examine the skyrmion dynamics for the current $j=|\varvec{j}|=0.006$ parallel to $\hat{\varvec{x}}$ under the condition $\alpha =\beta \ (=0.01)$ to avoid its current driven Hall motion.

**Figure 3:**
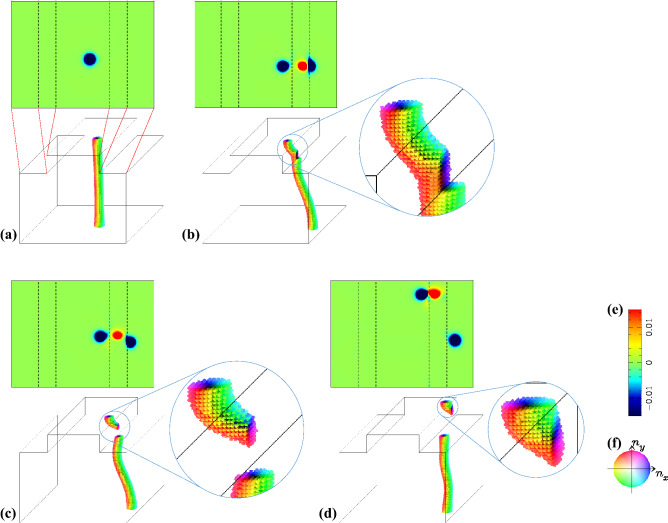
Pair creation and annihilation of the surface (anti)skyrmion(s). The height of the step edge is 20. (**a**) The upper (lower) panel represents the spatial distribution of emergent *b*-field $b_{\text {normal}}$ normal to the surface using color code (e) (magnetic texture using color code (f)) at $t=0$. The broken lines in the upper panel are corresponding to the edges of the upper and lower terraces as indicated by the red dotted lines. In the same way, the snapshots at (**b**) $t=4000$, (**c**) $t=4800$, and (**d**) $t=5400$ are shown. In (**b**–**d**), the enlarged images of the magnetic textures at around the right edge are also shown.

### Skyrmion number for surface magnetic texture

In this section, we first show the stability of the (anti)skyrmion at the surface, i.e., the surface (anti)skyrmion is not easy to annihilate even in the presence of the step edges of moderate height (the case summarized in Fig. 2). It is also shown by the conservation of the skyrmion number at the surface. Next, it is shown that the conservation of the skyrmion number at the surface applies for more complex dynamics where the skyrmion string is separated into pieces due to the large height of step edge (the case summarized in Fig. 3).

Figure 2 summarizes the current driven dynamics of the skyrmion string in the system with the step height 5. By the STT effect, the skyrmion string approaches to the left step edge (see Fig. 2a,b). However, the edge prevents the motion of the top endpoint of the string. Deep inside the magnet, the string moves by the STT effect, and gets bent and stretched as seen in Fig. 2b. After that, the top endpoint of the string, i.e., the top surface skyrmion overcomes the pinning due to the step edge and climbs up to the higher terrace as seen in Fig. 2a–c. After that, the skyrmion string shows a characteristic dynamics^40^ like ‘moving tornado’ reflecting the vorticity, Magnus effect and the tensile strain. (See also “Supplementary Information” and Movie S3.avi.) The current driven skyrmion string approaches to the right step edge and the top surface skyrmion at the higher terrace goes down to the lower terrace as seen in Fig. 2c–e. An interesting aspect of the dynamics is that the upper endpoint of the string always sticks to the surface even when the surface bents with $90^{\circ }$ at the step edge. Figure 2b,d actually shows the behaviors of the top surface skyrmion. (See also “Supplementary Information”).

This is understood to be the topological stability of the skyrmion at the surface: a way to define the topological nature of the string might be

6 $$\begin{aligned} N_{\text{ sum }}=\sum _z N_{\text{ topol }}(\Omega _z) \end{aligned}$$

with $\Omega _z$ being the horizontal plane at height *z* and ${\varvec{e}}=\hat{\varvec{z}}$. In the present case, there exist two regions with different heights of the top surface due to the step edges. Accordingly, $N_{\text{ sum }}$ changes along the dynamics, e.g., Fig. 2a–c and Fig. 2c–e. However, the change in magnetic texture along the dynamics shown in Fig. 2 occurs within a continuous deformation without topological singularity. Therefore, $N_{\text{ sum }}$ cannot be appropriate for the topological index for the magnetic texture. On the other hand, when we define the skyrmion number

7 $$\begin{aligned} N_{\text{ sk, } \text{ top }}=N_{\text{ topol }}(\Omega _{\text {top}}) \end{aligned}$$

with $\Omega _{\text {top}}$ being the developed top surface and ${\varvec{e}}$ points outward the magnet, it is confirmed that $N_{\text{ sk, } \text{ top }}$ is conserved during the dynamics summarized in Fig. 2. At the same time, it represents the topological protection of the magnetic texture at the top endpoint of the string. The topologically protected surface skyrmion dynamics is also well described by the time evolution of the spatial distribution of the emergent *b*-field $b_{\text {normal}}$ normal to the surface $\Omega _{\text {top}}$ which directly probes the deformation of the skyrmion (see the top panels of Fig. 2a–e). Although the skyrmion is strongly deformed due to the steep structure at step edges, the skyrmion keeps stick to the top surface during the dynamics. (See also “Supplementary Information”.)

We find that the surface topological index $N_{\text{ sk, } \text{ top }}$ is applicable for more complex phenomenon. Figure 3 shows the skyrmion string dynamics in the system with the step edges of height 20 and the string starts in the higher terrace area. Other conditions are the same as those for the case Fig. 2. By the STT effect, the string approaches the right step edge. Because of the repulsive interaction between the right step edge and the string, the string shows a bending behavior and touches the lower step corner first whereas its upper endpoint is still away from the step edge (see Fig. 3b). At the same time, at around the touched point, the magnetic texture of the string shows a deformation and finally the string splits into two parts, as shown in Fig. 3b,c. Note that the endpoint at the right step edge of the shorter string has a positive contribution to the topological index $N_{\text{ sk, } \text{ top }}$ whereas the contribution by the endpoint at the higher terrace surface is negative (see the plot of $b_{\text {normal}}$ in Fig. 3b–d). In other words, the emergent magnetic texture at the right step edge is the antiskyrmion. This causes a characteristic dynamics due to the topological nature^41,42^: after the skyrmion–antiskyrmion pair-creation shown as Fig. 3a–c, the skyrmion–antiskyrmion pair, i.e, the endpoints of the shorter string run together in $+\hat{\varvec{y}}$ direction as seen in Fig. 3c,d. The (anti)skyrmion has a vorticity and its sign is consistent with the sign of the topological index. Because of the vorticity, a Magnus force appears perpendicular to the force acting on the (anti)skyrmion^41,42^. In the present case, due to the tensile strain on the shorter string, an attractive force is acting on the endpoints of the string, i.e., the skyrmion at the higher terrace and the antiskyrmion at the right step edge of the top surface. Since the vorticity of the skyrmion is opposite to that of the antiskyrmion, the attractive force drives the Magnus force for the skyrmion and the antiskyrmion in the same direction. With this dynamics, finally, the shorter string disappears with the skyrmion–antiskyrmion pair annihilation at the top surface. Note that during the time evolution summarized in Fig. 3, $N_{\text{ sk, } \text{ top }}$ is conserved. This dynamics occurs without singularity of the magnetic configuration.

**Figure 4:**
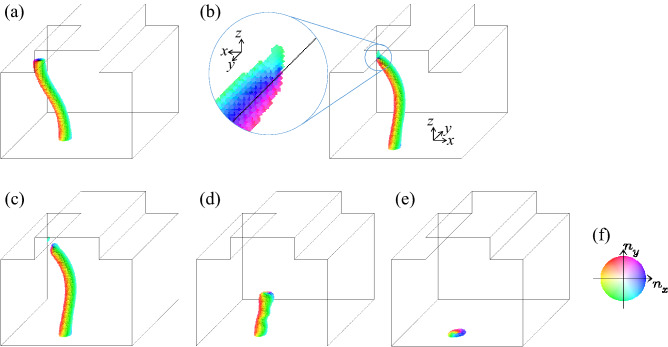
Monopole dynamics. The height of the step edge is 20. Snapshots of the magnetic texture at (**a**) *t* = 14,000, (**b**) *t* = 16,290, (**c**) *t* = 16,300, (**d**) *t* = 16,400 and (**e**) *t* = 16,480 are shown. (See text.) In (**b**), the enlarged magnetic texture is seen from left. (**f**) The color code for the magnetic texture.

**Figure 5:**
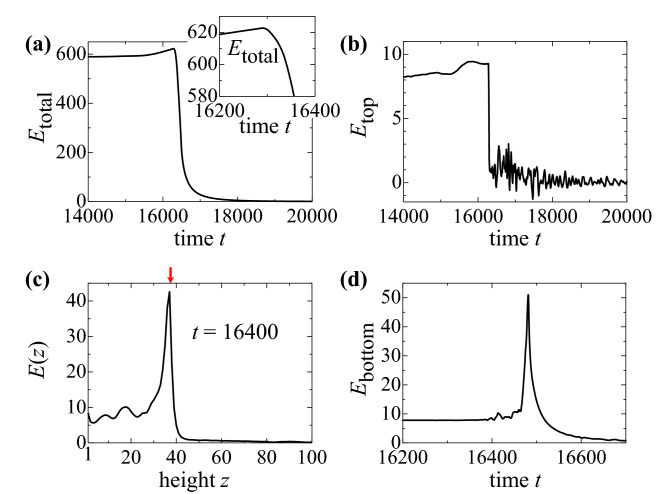
Monopole and singularity. (**a**) Time dependence of the total energy. The enlarged plot at around $t\sim$16,300 is also presented. (**b**) Time dependence of the energy of the top surface. (**c**) The local energy at height *z*. The red arrow indicates $z_{\text {mp}}$. The sharp decrease occurs at the time when the skyrmion is detached. (See text). (**d**) Time dependence of the energy of the bottom surface. The peak occurs at the time where the skyrmion on the bottom surface disappears.

### Creation and annihilation of monopole

In the present system, we can also discuss the magnetic texture with singularity of the magnetic configuration. Figure 4 summarizes the skyrmion string dynamics after those shown in Fig. 3. The STT effect drives the string in $\hat{\varvec{x}}$ direction. (Note that we impose the periodic boundary condition along *x*- and *y*-directions.) Similar to the dynamics in the initial stage shown in Fig. 2, the skyrmion at the upper endpoint of the string sticks to the top surface. (See Fig. 4a,b.) However, because the height of the step edge is high enough, the skyrmion string cannot overcome the barrier, and the skyrmion on the top surface is detached (Fig. 4c is the magnetic texture just after this ‘detach’ event.) After that, this upper endpoint of the string runs along the string and finally the string totally disappears. (See “Supplementary Information”.) After the upper endpoint of the string is detached, we find the topological discontinuity, i.e., the emergence of monopole: The monopole point ${\varvec{r}}_{\text{mp}}=(x_{\text{mp}},y_{\text{mp}},z_{\text{mp}})$ is an accumulation point where the magnetic moment is ill-defined. Therefore, it is not on the lattice site in $\Lambda$. Using $N_{\text {topol}}(\Omega _z)$, the topological discontinuity by $\varvec{r}_{\text{ mp }}$ is expressed to be, $N_{\text {topol}}(\Omega _z)=-1$ for $z<z_{\text{ mp }}$ and $N_{\text {topol}}(\Omega _z)= 0$ for $z>z_{\text{ mp }}$ and a relevant definition of $(x_{\text{mp}},y_{\text{mp}})$ will be given by the minimum of $n_z$ with an interpolated function on the the horizontal plane at $z=z_{\text{ mp }}$.

### Topological indices

The monopole charge is defined by $N_{\text{ mp }}=N_{\text {topol}}(\Omega )$ with $\Omega$ enclosing $\varvec{r}_{\text{ mp }}$ as discussed in the paragraph with Eq. (2). For the numerical results summarized in Figs. 2, 3 and 4, we find that the following relation always holds,

8 $$\begin{aligned} N_{\text{ mp }}=N_{\text{ sk, } \text{ top }}+N_{\text{ sk, } \text{ bottom }}, \end{aligned}$$

where $N_{\text{ sk, } \text{ bottom }}=N_\text {topol}(\Omega _{\text {bottom}})$ and $\Omega _{\text {bottom}}$ is the bottom surface with ${\varvec{e}}=-\hat{\varvec{z}}$. (See also Eq. (7).) Note that $N_{\text{ sk, } \text{ bottom }}=-N_{\text {topol}}(\Omega _z)$ with $z=1$ (see Eq. (6), and here, $z=1$ represents the bottom of the magnet). The domain of integral $\Omega$ enclosing $\varvec{r}_{\text{ mp }}$ is topologically the same as $\Omega _{\text {top}}+\Omega _{\text {bottom}}$. During the process shown in Figs. 2 and 3, $N_{\text{ sk, } \text{ top }}$ and $N_{\text{ mp }}$ are always zero. At the detach process of the top surface endpoint of the skyrmion string described in Fig. 4, the simultaneous changes $N_{\text{ sk, } \text{ top }}=-1\rightarrow 0$ and $N_{\text{ mp }}=0\rightarrow +1$ occur (and $N_{\text{ sk, } \text{ bottom }}=+1$ is kept).

For the dynamics summarized in Fig. 4, let us discuss the relation between the topological characteristics discussed above and the metastabilities of the magnetic textures, in more detail. Figure 5a shows the time dependence of the total energy $E_{\text {total}}$ measured from that of the relaxed ferromagnetic state. (See “Supplementary Information”.) Along the dynamics Fig. 4a,b, the total energy $E_{\text {total}}$ increases. This is because the upper endpoint is pinned by the left step edge and the string is bent and stretched by the STT effect. After that the total energy $E_{\text {total}}$ decreases rapidly with the detach of the top surface endpoint and successively occurring monopole dynamics Fig. 4b–d. At the detach process, the total energy $E_{\text {total}}$ shows rather smooth time dependence. The ‘hidden’ singular behavior along the emergence of the monopole is observed in the time dependence of the local energy at the top surface: we define the local energy on $\Omega$ by

9 $$\begin{aligned} E(\Omega ,t)=\sum _{\varvec{r}\in \Omega }\left[ E_t(\varvec{r})-E_f(\varvec{r})\right] , \end{aligned}$$

where $E_t(\varvec{r})$ ($E_f(\varvec{r})$) is given by Eq. (4) for the instantaneous magnetic texture at time *t* (for the relaxed ferromagnetic texture).

The plot Fig. 5b shows $E_{\text {top}}=E(\Omega _{\text {top}},t)$ as a function of time *t*. At around $t\sim$ 14,000, $E_{\text {top}}$ hardly changes because the skyrmion at the top surface is apart from the step edge. With approaching the skyrmion to the (left) step edge by the STT effect, the skyrmion becomes unstable due to its deformation. This causes the increase of $E_{\text {top}}$ and finally the sharp drop of $E_{\text {top}}$ occurs at the time when the skyrmion is detached, i.e., the emergence of the monopole. The increase of $E_{\text {top}}$ before the emergence of the monopole indicates the energy barrier dividing the skyrmionic state and ferromagnetic state at the top surface $\Omega _{\text {top}}$. The profile of the energy barrier seen in Fig. 5b is rather moderate compared to that in the discussion below because of the geometry with the step edge, i.e., the steep geometrical arrangements of the top surface reduce the metastability of the top surface skyrmion.

The singularity of the monopole is obvious in the local energy profile as shown in Fig. 5c. This plot shows the height $z \ (=1 \sim ~100)$ dependence of $E(z)=E(\Omega _z,t=$ 16,400). We clearly see the sharp energy peak which divide the metastable skyrmionic state and the ferromagnetic state. The red arrow on the top horizontal axis indicates the position $z_{\text {mp}}$, i.e., it divides the region of *z* by $N_{\text {topol}}(\Omega _z)=-1$ or 0. (See also “Supplementary Information” and Movie S8.avi.).

In Fig. 5a, after the monopole creation, the total energy $E_{\text {total}}$ decrease as a function of time *t* smoothly, i.e., no singular behavior is seen. This indicates a smooth motion of the monopole which makes the metastable skyrmion string shorter, although the monopole is a singular object as seen in Fig. 5c.

At the final stage, the collision of the monopole and the antiskyrmion occurs, and the monopole, the antiskyrmion on the bottom and the skyrmion string totally disappear with the simultaneous changes of $N_{\text{ sk, } \text{ bottom }}$ and $N_{\text{ mp }}$ from + 1 to 0. When we focus only on the bottom surface, we see the singularity with the energy cost: Fig. 5d shows the time dependence of the local energy at the bottom surface, $E_{\text {bottom}}=E(\Omega _{\text {bottom}},t)$. The sharp peak structure occurs with the simultaneous changes of $N_{\text{ sk, } \text{ bottom }}$ and $N_{\text{ mp }}$ from + 1 to 0. However, in the total energy $E_{\text {total}}$, this energy cost is compensated by the annihilation of the skyrmion string in total.

## Discussion and summary

The Gauss’ law Eq. (2) applies for the processes discussed in the present paper: suppose $\Omega$ is the whole surface of the magnet and the magnet has no spatial defects such as voids. There are two cases, (A) div $\varvec{b}=0$ in bulk and (B) div $\varvec{b}\ne 0$ in bulk.

- In case (A), the system has no (anti)monopoles. As shown in Fig. 3, the skyrmion string is divided into two within the continuous deformation of the magnetic texture. As a result, using the surface $\Omega$, any entanglements of the skyrmion string even in the presence of the knots, are solved without topological transitions. Therefore, it is concluded that any skyrmionic states are homeomorphic to each other and also those are topologically the same as ferromagnetic states and helix states in bounded three-dimensional magnets.
- In case (B), the system has (anti)monopoles. The (anti)monopole is a topologically singular object and cannot be created/annihilated within the continuous deformation of the magnetic texture. For a monopole-antimonopole pair, $N_{\text{ mp }}(\varvec{r}_{\text{ mp }})+N_{\text{ mp }}(\varvec{r}_{\text{ amp }})=0$ and it does not contribute to Eq. (2). Therefore, Eq. (2) is not appropriate to describe the topological invariance for the magnetic texture on the whole system.

To discuss the stability of the magnetic textures, the “local” monopole charge is important. The (anti)monopole always accompanies the high energy (being order of *J*) area concentrated at around $\varvec{r}_{\text{ mp }}$ ($\varvec{r}_{\text{ amp }}$). Consequently, for example, to break a skyrmion string into two at the point deep inside the magnet, for the monopole-antimonopole pair creation in other words, a large energy to overcome the energy barrier being order of *J* is required^40^. In this case, the change in absolute value $|N_{\text{ mp }}(\varvec{r}_{\text{ mp }})|+|N_{\text{ mp }}(\varvec{r}_{\text{ amp }})|$ is important rather than total monopole charge.

The energy cost at the (anti)monopole creation/annihilation is compensated by the shrinkage/deformation of the skyrmion string connecting the (anti)monopole as seen in Figs. 4 and 5. At the detach process of the skyrmion string from the top surface shown in Fig. 4b,c, we calculate the skyrmion number $N_{\text {sk,next-to-top}}=N_{\text{ topo }}(\Omega _{\text {next-to-top}})$ where $\Omega _{\text {next-to-top}}$ is the top surface of $\Lambda -\Omega _{\text {top}}$ ($\Lambda$ is the set of all sites $\varvec{r}$ of the system defined below Eq. (4)). We find a time duration with $N_{\text {sk,top}}=0$ and $\left| N_{\text {sk,next-to-top}}\right| =1$. This means that the monopole point $\varvec{r}_{\text {mp}}$ appears as an accumulation point between $\Omega _{\text {top}}$ and $\Omega _{\text {next-to-top}}$. Therefore, the monopole point $\varvec{r}_{\text {mp}}$ emerges without change of the length of the skyrmion string essentially, so that the energy cost due to the energy barrier discussed above appears in the time dependence of the total energy as seen in Fig. 5a. Even so, the sharp singularity due to the emergence of the monopole *point* is smeared in the total energy in three dimension.

In the present paper, we have seen the importance of the topological indices $N_{\text {sk}}$ and $N_{\text {mp}}$. These indices, specifically, are related by the Gauss’ law Eq. (2). In the previous studies^23,40^, it is discussed that the monopole dynamics running through the string causes the skyrmion string annihilation. The annihilation of a skyrmion string is seen in the final stage of the dynamics in Fig. 4, i.e., the collision of the monopole and the antiskyrmion at the bottom surface. On the dynamics, the skyrmion number at the bottom surface $N_{\text {sk,bottom}}$ changes from + 1 to 0. As seen in Fig. 5d, a steep enhancement of $E_{\text {bottom}}$ occurs with the change of $N_{\text {sk,bottom}}$. However, this enhancement of $E_{\text {bottom}}$ does not result in the protection of the bottom surface antiskyrmion. The energy cost by the local topological singularity seen in Fig. 5d is totally compensated by the energy gain due to the shrinking of the skyrmion string. Consequently, on the time window of this monopole-antiskyrmion collision dynamics, the total energy (see Fig. 5a) decreases smoothly and monotonously. Note that the changes of the topological indices $N_{\text {sk}}$ and $N_{\text {mp}}$ occur at the same time, and the Gauss’ law Eq. (2) always holds along the dynamics discussed here.

The skyrmion string annihilation instability is responsible for the (anti)monopole dynamics. For shorter skyrmion string, the probability of the emergence of the (anti)monopole(s) is reduced. This is why the skyrmion string is more stable in thinner magnets as has been observed experimentally^10^.

To summarize, we have discussed topological particles and strings on the magnets and their characteristic dynamics, e.g., particle-antiparticle pair creation/annihilation, collisions of the particles and behind string dynamics. To describe the dynamical processes of skyrmion string, (ant)skyrmion and (anti)monopole, we have shown that two topological indices, i.e., $N_{{\text{ sk }}}$ on the surface and $N_{{\text{ mp }}}$ in the bulk play the essential role.

## Methods

The units of time *t* is 1/*J*. Typically $J \sim 10^{-3}$ eV and the unit 1/*J* becomes $\sim$ 0.7 ps. The unit of the electric current density $j=|\varvec{j}|$ is $2eJ/(pa^2)$ and is typically $\sim 1.0\times 10^{13}$ A/m$^2$ for the polarization of magnet $p=0.2$ and the lattice constant $a=5$Å.

## Supplementary information

Supplementary Information.

Supplementary Video S1.

Supplementary Video S2.

Supplementary Video S3.

Supplementary Video S4.

Supplementary Video S5.

Supplementary Video S6.

Supplementary Video S7.

Supplementary Video S8.

## Data Availability

The data that support the findings of this study are available from the corresponding author upon reasonable request.

## Code availability

The original codes used in this study are available from the corresponding author upon reasonable request.
