# Supplementary material for: Bulk and surface topological indices for a skyrmion string: current-driven dynamics of skyrmion string in stepped samples
Source: Sci Rep. 2020 Nov 20;10:20303. doi: 10.1038/s41598-020-76469-5 (PMC7680146; doi:10.1038/s41598-020-76469-5)
Supplement: Supplementary file 1 — Supplementary Information. [file 41598_2020_76469_MOESM1_ESM.pdf]

Bulk and surface topological indices for a skyrmion string: current-driven dynamics of skyrmion string in stepped samples

Supplementary Information

Wataru Koshibae[1], Naoto Nagaosa[1,2]

[1]RIKEN Center for Emergent Matter Science (CEMS), Wako, Saitama 351-0198, Japan

[1,2]Department of Applied Physics, The University of Tokyo, 7-3-1, Hongo, Bunkyo-ku, Tokyo 113-8656, Japan

Interaction between step edges and skyrmion string

Figure S1 shows a chiral magnet with step edges: the height at the higher (lower) terrace is 10 (5). The other dimensions are the same as those for the system discussed in the main text. Numerical conditions are also the same as those used in the main text. In the initial stage, the skyrmion string is in the higher terrace area and close to the left step edge. The substantial in-plane component of magnetic moments appears near by the step edges due to the steep geometry. The in-plane component of magnetic moments at the step edge is not compatible with that of the skyrmion. Therefore, the skyrmion pushes away from the step edge.

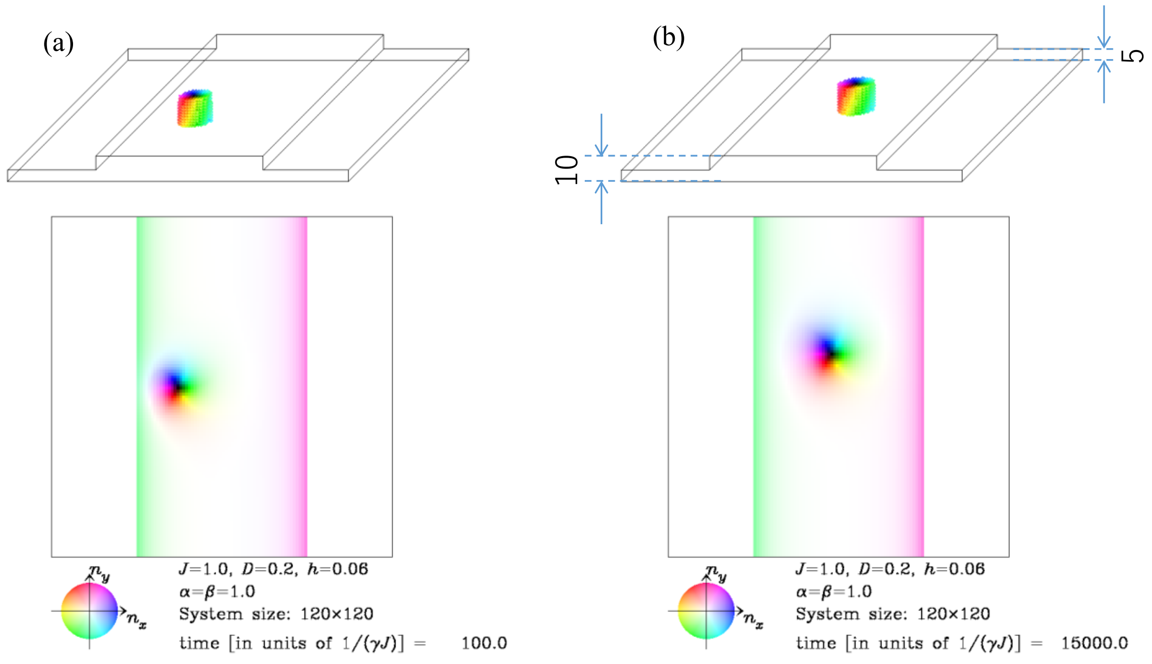

Fig. S1 (a) The magnetic texture at t=100 (see also the movie S1.avi). The upper (lower) panel shows the magnetic texture in a bird's eye view (in a horizontal cross section with z=10). The square by solid lines is a guide for eye for the in-plane system size. (b) The same as (a) but t=15000.

In Fig.S2, the skyrmion string is in the lower terrace area and has an enough distance from the left edge. In the present case, the in-plane component of magnetic moments at the step edge due to the

steep geometry is compatible with that of the skyrmion. As a result, the skyrmion approaches to the step edge in the relaxed state.

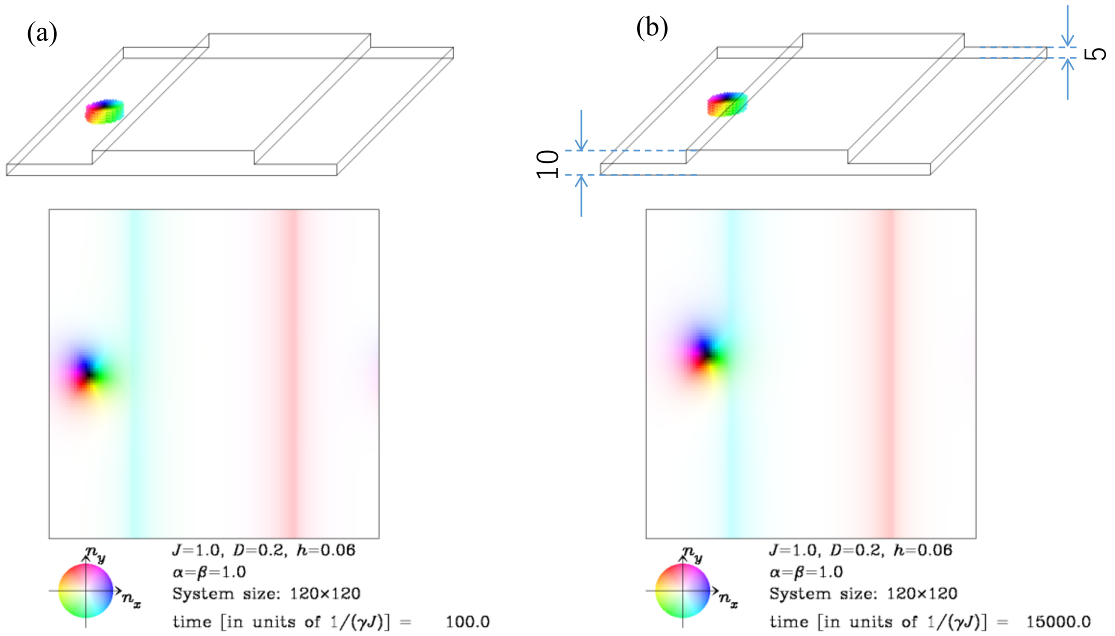

Fig. S2 The same as Fig.S1 but the skyrmion is in the lower terrace area.

Ferromagnetic ground state in the system with step edges

Figure S3 shows the relaxed ferromagnetic state in the system with step edges with a step height 20. Other conditions are the same as those in Main text. In the representation of the magnetic texture in main text and above section, the magnetic moments with $n_z > +0.5$ are not presented in the bird's eye view, but all the magnetic moments are presented here.

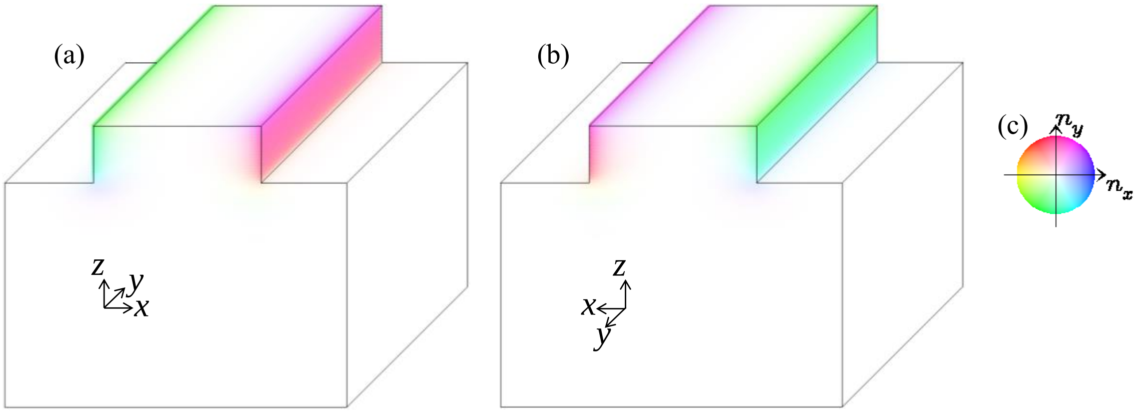

Fig.S3 Relaxed ferromagnetic state. (a) Magnetic texture using color code (c).
(b) The same as (a) but a view from left.

Cross sectional magnetic textures for Fig.4(d)

Figure S4 and movie S8.avi represent the cross sectional magnetic textures for Fig.4(d) at height z (=100~1).

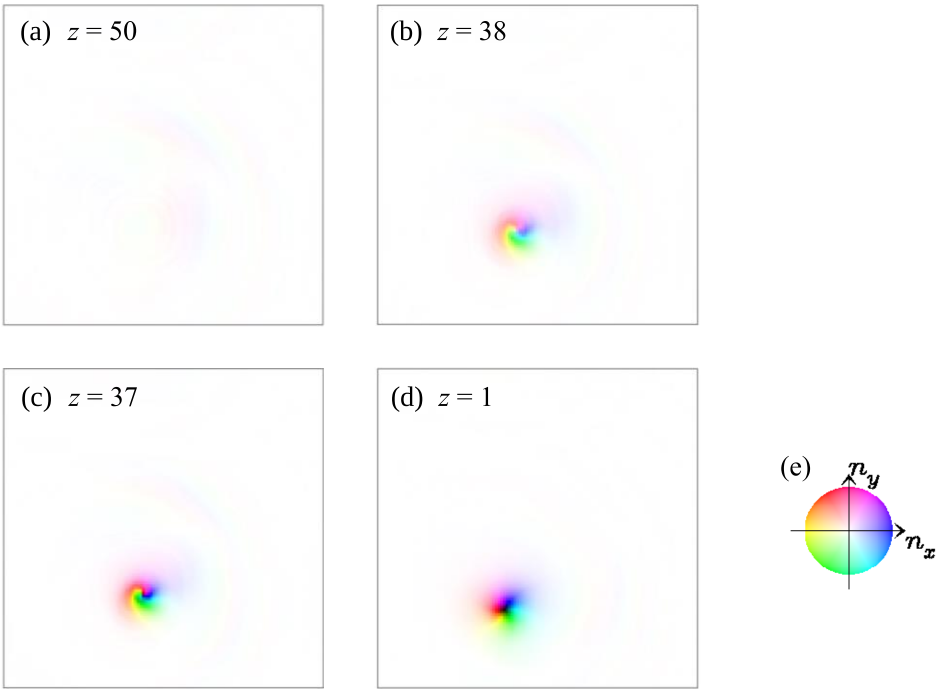

Fig. S4 Cross sectional magnetic textures (views from top) for Fig.4(d) at height (a) z = 50, (b) z = 38, (c) z = 37 and (d) z = 1 using color code (e). See also the movie S8.avi.

Supplementary Movies

S1.avi: Movie for Fig.S1.

S2.avi: Movie for Fig.S2.

S3.avi: Movie for Fig.2. (Magnetic texture)

S4.avi: Movie for Fig.2. (b-field at top surface)

S5.avi: Movie for Fig.3 and Fig.4. (Magnetic texture)

S6.avi: Movie for Fig.3 and Fig.4. (b-field at top surface)

S7.avi: Movie for Fig.4. (Magnetic texture)

S8.avi: Movie for Fig.4(d). The magnetic textures (the views from top) at height z (=100~1).
